# Development of pedagogical knowledge among learning assistants

**DOI:** 10.1186/s40594-017-0097-9

**Published:** 2018-01-08

**Authors:** Laken M. Top, Sarah A. Schoonraad, Valerie K. Otero

**Affiliations:** School of Education, University of Colorado Boulder, Boulder, CO 80309 USA

**Keywords:** Learning assistant, LA, Pedagogy, Knowledge construction, Formative assessment, Students’ ideas, Misconceptions, Course transformation, Course reform

## Abstract

**Background:**

Successful outcomes of the Learning Assistant (LA) model include increased learning outcomes in STEM gateway courses and increased persistence to graduation among LAs and the students they serve. While there are many possible reasons that the LA program is effective, the pedagogical development of the LAs themselves has not yet been systematically studied. The research reported here investigated how deeply first-time LAs enrolled in a one-semester pedagogy course took up the language associated with the course’s essential pedagogical principles. By reviewing prior research as well as assessing our target population and our pedagogy course learning goals, we developed a set of three essential pedagogical principles that are critical for effective classroom instruction and developed a coding scheme for identifying these principles in LAs’ written work. We then looked at LA’s development of language with respect to these principles by analyzing weekly teaching reflections submitted by LAs during five iterations of our pedagogy course.

**Results:**

Our research indicated that the language used to introduce particular pedagogical principles might play an important role in initiating LAs’ uptake of these concepts.

We found that LAs began to develop an understanding of the language that values students’ prior ideas in learning, but the depth of this understanding varied. In addition, LAs did not demonstrate as much growth in their language with respect to the formative assessment or to the idea that students play a role in constructing knowledge.

**Conclusions:**

In developing a pedagogy course for LAs, relating to their prior backgrounds in STEM appears to be critical. Using language that is accessible seems to increase LAs’ ability to develop pedagogical principles. Although LAs’ development of language related to the essential pedagogical principles is small, it may be enough to allow them to create contexts that facilitate learning.

## Background

Learning Assistants (LAs) are undergraduate STEM majors who are hired to assist faculty in making STEM courses more interactive and student-centered. In addition to meeting weekly with the lead instructor of the course and facilitating group interactions among enrolled students, LAs take a special pedagogy course intended to introduce them to relevant pedagogical principles and practical, research-based instructional strategies. LAs’ work with students, typically in large-enrollment, introductory courses, can be thought of as the field-based component of the pedagogy course. As instructors of the pedagogy course, we would like an efficient way to measure the extent to which LAs learn the content of the course; however, the literature is sparse with respect to the assessment tools for measuring learning of pedagogical principles—both within higher education and in the teacher education literature. The intent of this paper is to attempt to measure the extent to which LAs understood the pedagogical content presented in the pedagogy course. In standard STEM courses, an end of the semester comprehensive exam is often used to assess whether students learned the main content of the course. Even more acceptable over the past decade or so are pre/post-conceptual assessments that allow instructors to measure learning gains—how much students learned that they did not already know (NRC 2013). The work reported here outlines a method for measuring LAs’ learning of pedagogical principles by investigating pre- and post-semester written work. We describe a relatively easy-to-use coding scheme and use it to analyze the written pre/post-reflection assessments that are assigned in the pedagogy course. This “learning gain” measure is intended to shed light on the broader question of the role of pedagogical knowledge in LAs’ effectiveness.

The LA program has been found to be effective in multiple ways. There is strong evidence, for example, that LA-supported courses lead to greater learning outcomes among students enrolled in the course (Pollock 2009; Nelson 2011; Talbot et al. 2016), that LAs develop content understanding and identities (Close, Conn, Close 2016), and that LAs recruited to K-12 teaching exhibit more reform-based teaching practices than their peers (Gray, Webb, and Otero 2016). Recent work shows that having one LA-supported course increases the likelihood of graduating by 6% for all students and by 15% for first-generation college students (Alzen, Langdon, and Otero, in press). Other recent work demonstrates that the probability of completing an introductory STEM course with a grade of C or better is increased by 4 to 14%, with the greatest impact on students from groups traditionally underrepresented in STEM disciplines (Alzen, Langdon, and Otero, in press). Also, LAs themselves show a 10% increase in persistence to graduation over a matched sample of their peers (Otero 2015). The effectiveness of the LA program is likely due to many factors ranging from students’ interactions with LAs to faculty and institutional change. Here, we focus only on the role of LAs’ pedagogical learning.

In this study, we investigated the research question: To what extent did LAs refer to essential pedagogical principles in written responses to questions about teaching and learning? We also investigated the sub-question of how deeply the language of certain pedagogical principles was taken up by LAs when posed with challenging, abstract questions about these concepts.

## The learning assistant program

The LA model is a model of institutional change (Goertzen, Brewe, Kramer, Wells and Jones 2011; Otero 2015), which seeks to impact institutional values and practices through a low-stakes, bottom-up system of course assistance by awarding LAs to faculty and courses and by empowering faculty and departmental coordinators to institute instructional change. The Learning Assistant program intends to improve the conditions for learning particularly in large-enrollment, undergraduate courses and to recruit math and science majors to careers in teaching. While there are variations among programs throughout the world and within departments at a single university, the LA programs ideally consist of three main components, namely LAs: (1) interact with groups of students, (2) engage with faculty members in weekly preparation meetings, and (3) participate in a pedagogy course designed for new LAs. In their interactions with students, LAs facilitate discussions among students enrolled in courses about conceptual problems within various science, math, and engineering disciplines. LAs focus mainly on eliciting student thinking, helping students engage in discussions to extend and revise their initial ideas, and helping students in groups develop shared understandings of the content of the course. In their weekly preparation meetings with the faculty member(s) leading the course, LAs develop deeper content understanding and exchange information with course instructors about how students are engaging with course content (Otero 2015). In these weekly preparation sessions, they often reflect on the previous week, plan for the upcoming week, and analyze assessment data. All LAs also take a 15-week pedagogy course throughout their first semester serving as LAs. The pedagogy course introduces LAs to the educational research literature, learning theory, and strategies that support eliciting student ideas within STEM disciplines and using these ideas to guide questioning and actions that can help individuals in groups work together to make sense of a topic. The pedagogy course also addresses topics such as building relationships with students, mindset, metacognition, and differentiation. This paper focuses specifically on the language used by LAs that provided insight into their understanding of students’ ideas and the role of these ideas in facilitating learning. Although not all LAs intend to become teachers, the program is designed to help them learn effective practice through their LA experience.

The LA program began on our campus in 2001 with eight LAs in one department. Currently, on our campus, 380 LAs are hired by 13 STEM departments each year to work with approximately 90 faculty members. The model has also spread throughout the world, with programs in Singapore, Japan, India, Canada, and Ireland. According to the Learning Assistant Alliance data capture tool, there are 99 active programs globally using 1042 LAs each year in 295 departments, with 306 faculty members. The LA program is generally designed to address the learning of multiple populations including the LAs themselves, the undergraduate students with whom they work, and the faculty members who use LAs. The current study focuses only on the learning of the LAs themselves, specifically on what they learn about certain pedagogical principles.

In the pedagogy course, LAs are introduced to selected pedagogical principles, which they then practice with students. Most LAs enter the LA program strongly identifying with science, math, or engineering disciplines and with little intention of pursuing a future career in education. Thus, a course on pedagogy must connect with LAs’ past experiences and their future goals. Furthermore, it is necessary that care is taken to distill and focus the pedagogical content that LAs are expected to develop and practice in a 15-week time period. Although the LA program is, by design a practice-based experience for LAs, the focus of the pedagogy course is on *learning* rather than on teaching. We have found that LAs are more sensitive to the needs of learners when they are positioned in a peer-peer relationship (Otero 2005). For this reason, the pedagogy course attempts to avoid the *teaching stance* (Levin, Hammer, and Coffey 2009) which would imply a hierarchical relationship between LAs and the students whom they assist.

## Conceptual framework: essential pedagogical principles

Historically, many attempts have been made to distill, list, and define central pedagogical principles such as high-leverage teaching practices (Windschitl, Thompson, Braaten, and Stroupe 2012), constituents of active learning (Meltzer and Thornton 2012), and core teaching practices (Kazemi, Lampert, and Franke 2009; Ball and Cohen 1999). These consolidations have been influenced by time period, audience, changing language preferences, and theoretical foundations of particular teacher preparation programs (Zeichner 2012), yet their essence has remained relatively consistent. In developing the content that would be addressed in a pedagogy course suitable for the specific population of science, mathematics, and engineering majors who serve as LAs, we extracted the essence of effective pedagogical practices from the literature. Assumptions and generalizations had to be made in order to establish a small set of *essential pedagogical principles* that could be addressed in the LA pedagogy course. Just as a simplified “three-layer earth model,” which generalizes the earth as composed of three layers—core, mantle, and crust—is useful for understanding a wide variety of complex earth systems; a generalized set of essential pedagogical principles can be useful for helping researchers understand a variety of complex learning outcomes. The first essential pedagogical principle or “core” of our model derived from the literature is the principle that students’ ideas are important for instruction. By this, we mean the class of information that acknowledges the importance of what students bring into the classroom with them, that is their prior knowledge, ideas, or experiences related to a given topic. We will use the abbreviation *students’ ideas* to refer to this principle in the rest of this manuscript. The “mantle,” the principle of *constructing knowledge*, helps build a framework of learning in which students are actively engaged in building understanding, and the “crust”, the principle of *formative assessment*, looks at how instructors can facilitate and leverage students’ ideas with respect to an instructional goal.

Fully acknowledging that these essential pedagogical principles are by no means mutually exclusive or all encompassing, we hold that they are widely accepted and generalize a wealth of available scholarship on what constitutes effective classroom instruction. While the generalized grouping of literature into three broad categories will certainly ignore various nuances in specific scholarly areas, there is value in making such approximations analogous to how the three-layer earth model has supplied scientific insight in seismic waves, plate tectonics, and magnetic fields. We explain the three pedagogical principles in more detail below.

### Student ideas

The notion of students’ ideas as influential in instruction has appeared in both K-12 and higher education literature in many forms: student conceptions and difficulties (McDermott 1984; Feher and Rice 1988), naïve theories (McCloskey 1983), expert/novice differences (Chi, Feltovich, and Glaser 1981), and mental models (Gentner and Stevens 1983; Redish 1994) to name a few. According to the vast literature, students’ ideas may be derived from prior classroom instruction or they may be an amalgamation of life experiences, or some combination thereof. Researchers generally agree that students’ ideas act as a filter for concepts, symbols, and formalisms presented through schooling and that they affect the way that students engage with new ideas (NRC 2012). Curriculum developers throughout the 1980s and 1990s developed numerous subject-specific indexes (e.g., AAAS 1993; Driver, Guesne, and Tiberghien 1985; Driver, Squires, Rushworth, and Wood-Robinson 1994) which generally focused on how students’ ideas can create difficulties for learning new concepts. Although this perspective has been valuable for curriculum and instruction, it is also somewhat limited in scope. By the 1990s, students’ ideas were represented in the literature as robust learning resources that could be further developed through instruction (Hestenes 1987; diSessa 1988; Clement, Brown, and Zietsman 1989; Minstrell 1991; Smith, diSessa, Roschelle 1994; diSessa and Sherin 1998). Researchers coupled the notion of students’ ideas as resources with *framing* (Tannen 1993) in order to discern how students’ articulations are both context-dependent and highly sensitive (Hammer, Elby, Scherr, and Redish 2005). More recently, the resources framework has appeared as “conceptual entities” (Manz 2012) and “students’ diverse sense-making repertoires” (Rosebery et al. 2015). Regardless of terms used, researchers collectively acknowledge the value of using students’ ideas for effective teaching and learning (Meltzer and Thornton 2012).

### Constructing knowledge

The second essential pedagogical principle—*constructing knowledge*—goes beyond acknowledging that students have useful ideas and foregrounds instead how these ideas are reorganized and articulated as student generate new knowledge. Since the mid-1800s (e.g., Wead 1884; Dewey 1910), educational literature and national reports consistently called for instruction that provides opportunities for students to induce principles about observed phenomena through laboratory experiences and refining and building upon their prior ideas in the face of new data (AAAS 1993; NRC 1996; NRC 2012; NGSS 2013). In the 1980s, the constructivist epistemology (Glasersfeld 1989) was a popular perspective used by researchers to understand how learning was based on prior knowledge. This perspective used the biological notion of *viability* to explain how learners construct knowledge by reconciling new experiences and ideas with prior constructs to develop models that “fit” within a given situation.

Similarly, the notion of “existing conceptual schemes” (Taber 2001) proposes that learners “re-construct” their understanding of concepts by using prior knowledge to decipher new information (Taber 2013). Other terms such as “inquiry,” “discovery learning” with scaffolding, and “guided inquiry” have been used to refer to the process of constructing knowledge from old and new experiences (Klahr 2000; Hogan and Pressley 1997; Linn, Bell, and Davis 2004; Quintana et al. 2004; Reiser 2004; Sherin, Reiser, and Edelson 2004). Some researchers have appealed to the classic work of Vygotsky (1986) to illustrate how learners’ ideas and experiences interact with terms and concepts presented through schooling, leading to conceptual development (Otero and Nathan 2008). More recently, Rosebery et al. (2015) use the term “intellectually generative” to refer to the value of students’ ideas to construct knowledge.

### Formative assessment

The third essential pedagogical principle—*formative assessment*—refers to the activities that a teacher carries out in a classroom environment that encourage students to construct knowledge on the basis of their prior ideas and experiences. These teacher actions consist mainly of the teacher taking stock of students’ ideas, setting a goal with respect to a given topic, and making decisions about the best way to help a student move from their current position to where the teacher would like them to be. This process has been illustrated as a bridge (Forzani 2014; Atkin, Black, and Coffey 2001) that begins with students’ previous knowledge, involves questioning and other interactions between teacher and student, and ends with a more developed or refined understanding of a particular concept. The literature on formative assessment is vast and characterizes it in many ways. In a review, Gray (2013) presented formative assessment as a spectrum beginning with formative assessment as teaching strategies that use feedback (Kluger and DeNisi 1996) to strategies that focus on student ideas, goals, and instruction (e.g., Ruiz-Primo and Furtak 2006; Shepard 2005) and to formative assessment as a philosophical stance on learning (Lave and Wenger 1991). Building on the work of Ball and Cohen (1999), Rosebery et al. (2015) represent formative assessment as *interpretive power*, through which teachers assess students’ responses as nuanced and refined rather than as simply correct or incorrect, and adjust instruction accordingly. Although Rosebery’s approach includes a strong focus on differentiation and equity in the classroom, at their core, both formative assessment (in its numerous instantiations) and interpretive power are about listening to and valuing students and adjusting teaching approaches accordingly with respect to a goal.

Although there has been variation in language and subtle changes in application throughout the years, researchers and teacher educators seemingly agree on the essence of various pedagogical principles: student ideas, constructing knowledge, and formative assessment. These components of effective classroom instruction seem to apply across a wide variety of learning environments, including those in which LAs participate.

## Difficulty learning pedagogical principles: conflicting experiences

Despite the abundance of research that agrees upon a handful of general pedagogical principles, there remains an infrequent application of these principles at all levels of education (NRC 2013; Davis 2003; Weiss 1997). This has been the case for over 130 years in science education as examined by Meltzer and Otero (2015) and Otero and Meltzer (2017) in their historical accounts of physics education reform. This work suggests that helping course instructors learn essential pedagogical principles in practical ways is a great challenge. Furthermore, literature that provides evidence regarding the extent to which pedagogical principles are actually learned is also scarce. We explore some of this literature here and note that while this review represents a range of research from higher education to teacher preparation programs, the findings are very informative for studies involving LAs who play a unique, multifaceted role in the university setting. Not only are LAs students of pedagogy, allowing them to share similarities with pre-service teachers, they are also involved in higher education settings, working closely with college faculty, instructors, and teaching assistants. Thus, educational research from a broad spectrum of the literature can be used to guide studies involving LAs.

Weiss (1997) revealed that even though reforms in US standards of the day emphasized student engagement rather than lecture-only methods, up to 94% of high school math and science classrooms continued to use the lecture format. Of those, up to 90% indicated that they believed students learned best when students are actively engaged. This trend exists at the university as well. A committee on physics education (NRC 2013) reported that although there exists a wealth of research-based materials and tools for undergraduate physics, these tools and strategies are not broadly implemented. The dominant form of physics classroom instruction continues to be a lecture, not well aligned with the essential pedagogical principles described above.

Research has shown that there are contextual and other factors explaining the dearth of agreed upon practices in actual classrooms (Meltzer and Otero, 2014; Henderson and Dancy, 2007; Dancy and Henderson 2010; Prince 2004; Powell 2003; Borg 2004; Davis 2003; Lortie 1975). The literature further suggests that some of these contextual factors differ between disciplines within a single university setting (Lund and Stains 2015; Shadle, Marker, and Earl 2017). It has also been argued that essential pedagogical principles are simply difficult to learn. Smagorinsky, Cook, and Johnson (2003) argue that teachers leave their teacher education programs with a “pseudoconcept” for teaching that must be further developed through many years of professional practice, which is socioculturally nuanced and authentic. Otero and Nathan (2008) showed that only 28% of prospective teachers in their study demonstrated a complete understanding of the formative assessment process the final semester before completing the teacher preparation program. These low improvements might be explained by the role of prior experiences and prior concepts about *teaching* with which prospective teachers enter their pedagogy instruction.

Just as the students we teach in science classes come in with prior ideas about the natural world, teachers come into their learning environments (pedagogy course, methods courses, etc.) with a number of pre-existing ideas about teaching and learning based on their experiences (Borko and Putnam 1996). These ideas or beliefs that educators hold may not be entirely known to them, and yet they act as a filter and impact their understanding of pedagogy (Davis 2003; van Dreil and Verloop 2002). Lortie (1975) suggested that because prospective teachers have had many years of experience participating as students in courses not well aligned with essential pedagogical principles, it can be very hard for them to adopt a different, often contradictory, view of teaching in only a short time of instruction. Consistent with the *students’ ideas* pedagogical principle, teachers’ previous ideas about instruction act as a filter for concepts and practices presented in pedagogy or other teaching method courses. Furthermore, LAs often find themselves in environments that offer conflicting views on teaching and learning than those offered in the pedagogy course. We have found that there can exist a conflict between the pedagogical philosophies presented in the pedagogy course and those held by the professors and the teaching assistants of the disciplinary courses. Although professors have presumably applied to the LA program and are interested in the benefits afforded by incorporating LAs into their classrooms, they often have limited experience with progressive educational pedagogy and may therefore still have ideas about teaching and learning that diverge from the principles we are trying to help LAs develop. In addition, our experience has shown that many graduate students in STEM fields are not heavily invested in undergraduate education and hence, working with graduate teaching assistants may also influence LAs’ views on teaching and learning in a way that conflicts with the essential pedagogical principles that we have proposed. Thus, we expect that LAs may demonstrate small amounts of growth with respect to pedagogical ideas within a single semester. We are aware of few studies, however, that actually measure learning outcomes involving pedagogical principles in terms of changes in thinking from pre-instruction to post-instruction. Much of the literature on teacher learning focuses on the difference between what teachers say they do and what they actually do in practice (e.g., Henderson and Dancy 2007; Crawford 2007; Eimers et al. 1998; Weiss 1997) rather than on what teachers and prospective teachers learn (as measured by written assessments) from their courses or programs that focus on pedagogical principles.

In the current study, we examine pre/post-measures of LAs’ language related to the essential pedagogical principles. We examined 1206 questions from 304 LAs’ reflections from 13 departments in an effort to understand the extent to which the language related to essential pedagogical principles was taken up by LAs.

## The LA pedagogy course

LAs who take the pedagogy course are undergraduate students who typically (a) are serving as LAs for the first time; (b) are second-semester freshmen through seniors; (c) are primarily majoring in math, science, engineering, or psychology/neurosciences; and (d) have not explicitly considered teaching as a career option. In efforts of embracing LAs’ identities, interests, and experiences, the pedagogy course must focus specifically on the practicalities of their current and past experiences while still addressing the essential pedagogical principles that it promotes.

There were four to six sections of the pedagogy course each semester throughout the duration of the study. Each section consisted of LAs from a variety of disciplines, though attempts were made to group LAs from complimentary departments. Instructors included university faculty, upper-division science and math education graduate students, and clinical faculty (former K-12 teachers). All pedagogy instructors had experience teaching at some level. The three authors taught the pedagogy course at least one semester that was studied.

### Course design

The pedagogy course design has always incorporated the three pedagogical principles with minor variations in the resources used to introduce them. During the semesters included in the current study, the organization of each pedagogy class session typically began with a discussion of an article the LAs were expected to read, usually supported by a set of planned discussion questions. This was generally followed by a group-based activity designed to assist LAs in using and processing the concepts addressed in the paper. Such activities included immersion exercises in which LAs were engaged as students in a model science lab activity illustrating one or more of the essential pedagogical principles or a video-based activity in which LAs analyzed student-student interactions or made inferences about students’ prior ideas within a particular content area. Activities were often followed by debriefs intended to match the activity with the concepts addressed in the literature and how it aligned with LAs’ experiences working with students. Examples of different types of class activities are provided below. We note that although the activities below were implemented in weeks specifically highlighting each of the pedagogical principles, assignments and discussions carried out in weeks of the semester with other “focus” topics also sought to weave in and emphasize these essential principles as they were foundational for all concepts that were introduced in the course.

### Student ideas

The terminology “mental models” was one of the terms used to address the pedagogical principle of students’ ideas in four of the five semesters studied. Using classic cognitive science references, Redish (1994) argues that students’ have pre-existing mental models and that educators must use them to guide instruction. Admittedly, this framework does have weaknesses including the perception of mental models as large, difficult to change, and irreducible. Despite these limitations, we have found the Redish article to be impactful for LAs, who often have not considered that pre-existing mental constructs can influence learning math and science content. Redish (1994), which is used early in the semester, is written by a physicist in a format that is much like a proof in mathematics; it uses axioms and corollaries to convey information about mental models and other learning theory, making it accessible to LAs. Our informal observations of LA class discussions have lead us to believe that stylistic and language practices used in this article are effective in tapping into LAs’ science and math identities while at the same time connecting them to the literature from cognitive science and learning theory. In all instantiations of the course, LAs participated in activities designed to help identify and discuss the value of the student ideas principle within a disciplinary context. For example, in one activity, LAs watched a video segment of 4th-grade students tasked with determining which objects from a given set would sink or float and to determine a set of “rules” (principles) for what caused things to sink or float. By watching the video, LAs identified valuable ideas in 4th graders’ thinking, such as “objects that float have air in them” and “objects sink because they are heavy for their size”.

### Constructing knowledge

**Fig. 1:**
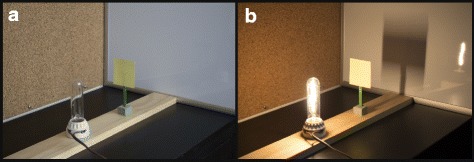
First stage of the light experiment. Experimental set-up from which LAs make predictions (**a**), observation of complex shadow, different from the sharp square image that LAs typically predict (**b**)

Although the essential pedagogical principle—*constructing knowledge*—has always been a part of the pedagogy course, readings that highlighted the term “constructing knowledge” or “constructivism” were only present in the Spring 2016 semester. An activity that took place throughout the study to demonstrate the concept of *constructing knowledge* involved LAs’ being immersed in a content-specific activity on geometric optics. In this laboratory activity, LAs participated as students in constructing the idea of ray diagrams and constructing the idea that a continuous light source could be thought of as an infinite sequence of point sources. The activity began by eliciting LAs’ initial ideas about an experimental set-up involving a continuous light source, a blocker, and a screen (see Fig. 1a). They then observed the complex shadow (see Fig. 1b) and were tasked with developing an explanatory model for why the complex shadow appeared as it did.

**Fig. 2:**
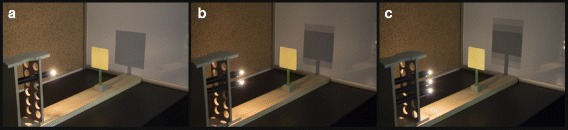
Second stage of light experiment. Observation using pedagogical tools: one Mini-Maglite™ (**a**), two Mini-Maglites™ (**b**), and three Mini-Maglites™ (**c**)

After making the observation, LAs were provided with several point sources (Mini-Maglites™) and a stand to arrange them in order to reproduce a discrete version of the complex shadow they observed so that they could construct an explanatory model and invent the ray diagram (see Fig. 2c).

Experiencing first hand, an activity that involved a complex phenomenon and provided the pedagogical tools (Mini-Maglites™) for constructing knowledge on their own was useful for helping LAs understand and believe the role that engagement with tools, scaffolding, and groups plays in learning. This activity and the subsequent group discussion also highlighted the valuable (and problematic) role that students’ prior ideas play in the learning process.

### Formative assessment

The concept of formative assessment has been included in the pedagogy course since its inception, however, the term formative assessment was only introduced through readings in 2009. For example, LAs identified a learning goal, considered students’ ideas with respect to that goal, and crafted an instructional intervention that moved students from where they are to where we want them to be. Currently, LAs read Moss and Brookhart (2010), which is relatively accessible to them. This chapter describes formative assessment as an assessment *for* learning rather than an assessment *of* learning, suggesting that there are three guiding questions to the formative assessment process, (1) “Where am I going?,” (2) “Where am I now?,” and (3) “What strategies can help me get to where I need to go?” In the pedagogy course, LAs constructed a bridge on an actual diagram by articulating common ideas they had observed among their students, specific daily learning goals for the course in which they were an LA, and the types of questions that might help students make progress in their thinking from their initial idea toward the learning goal. Like the concept of constructing knowledge, formative assessment is woven throughout the pedagogy course as LAs are introduced to practical techniques for using students’ ideas to construct knowledge and shared understandings.

## Methods

### Participants

The participants of this study were 304 first-time LAs who were enrolled in the LA pedagogy course in one of five semesters, Spring 2014 to Spring 2016 from 13 departments, primarily working in science, mathematics, engineering, and psychology courses. The distribution of LAs by department and semester is shown in Table 1. Note that departments represent the courses that LAs worked in, not necessarily their majors.

**Table 1:** Distribution of the study participants by semester and by department

	Spring 2014	Fall 2014	Spring 2015	Fall 2015	Spring 2014	Total
Applied Mathematics	8	15	3	8	5	39
Astronomy	7	2	5	3	5	22
Atmospheric Sciences	4	2	4	5	4	19
Chemistry	17	8	13	20	11	69
Engineering	0	1	0	0	0	1
Evolutionary Biology	5	2	5	3	7	22
Education	4	2	4	2	2	14
Geology	4	0	0	0	0	4
Mathematics	2	3	9	7	9	30
Molecular and Cellular Biology	5	8	2	6	1	22
Neuroscience and Psychology	1	3	6	7	3	20
Physics	11	10	7	5	9	42
Total	68	56	58	66	56	*N* = 304

All first-time LAs who completed both the first and last teaching reflection of the semester (described below) were selected to be part of our analysis. Table 1 includes only LAs who completed both first and last reflection. Due to our focus on LA growth, it was important for us to have pre/post-responses from each LA in the study. A total of 304 LAs out of 455 participated in the study.

### Teaching reflections

Teaching reflections have been assigned in the LA pedagogy course consistently since 2002 and provide a way for LAs to reflect on their experience and thoughts about the pedagogical topics discussed. LAs are asked to write weekly responses that reflect on their opportunities to implement the pedagogical strategies in their LA sessions, any concerns, questions, or triumphs they might be experiencing, and their ideas about what teaching, learning, and being an LA means to them.

In all semesters in this study, LAs have been asked identical questions in the second and 15th week of the semester, which serve as pre/post-views. Spring 2014 is the only exception, with the “post” questions being asked in week 13 instead. We found that the Spring 2014 data was similar in nature to that of the other semesters analyzed. We concluded that the two-week disparity did not influence students’ responses. The two reflection questions that we were interested in were “What does it mean to teach?” and “What does it mean to learn?,” hereafter referred to as the teach prompt and the learn prompt. We chose these questions because they had the potential to provide insight into how LAs viewed students with respect to the teaching/learning process and whether they would be able to articulate the essential pedagogical principles without being asked directly. We investigated the research question: To what extent did LAs refer to essential pedagogical principles in written responses to questions about teaching and learning? We also investigated the sub-question of how deeply the language of the pedagogical principle of students’ ideas was taken up by LAs. All LAs that participated in the study responded to both teach prompts and both learn prompts for the semester. The only exception to this was in Spring 2016 when five LAs completed the pre/post-teaching questions, but not the learning questions because they were permitted to choose two of four questions. Overall, we analyzed 1206 teaching reflections across the five semesters.

### Coding and analysis

Three essential pedagogical principles that were learning goals for the pedagogy course were student ideas, constructing knowledge, and formative assessment; hence, these are the focus of our methodological approach to measuring LA growth in their first semester in their LA role. Our analytical approach involved descriptive coding using a priori codes representing *student ideas*, *constructing knowledge*, and *formative assessment*. Although we took a deductive approach to coding, we did not initially compose formal definitions for each of our a priori codes. Rather, the researchers coded the responses to the teach and learn prompts separately, first relying on their own conceptions of what these codes meant. The responses to each of the prompts were coded holistically, that is, each response could be coded at most once for each of the three principles. Then, the researchers came together to compare codes and developed formal and concrete definitions (Table 2) before re-analyzing all responses using the analytical method of simultaneous coding (Miles, Huberman, Saldaña 2014). We felt that this was appropriate because while the essential pedagogical principles are distinguishable, they are not mutually exclusive, and we found that LA responses could include some aspects of one or all of the principles. Furthermore, since we were studying LA growth in learning, it made sense that their responses might not be a clear expression of just one thought, but that they might be an amalgamation of different notions.

**Table 2:** Summary of primary codes used to analyze LA teaching reflections

Code	Definition	Examples
Student ideas	Acknowledgement of at least one of the following: • Students come in with ideas (using pedagogy vocabulary, e.g., mental models) • Prior ideas could impact the way the new information is taken up • These ideas could be built upon/related to when teaching new information • The new information may cause some rearrangement of a pre-existing framework	“To help them when they got stuck in their mental models” (Spring 2015) “…what the individual knew before.” (Spring 2014) “A teacher works with what the student thinks.” (Spring 2016)
Constructing knowledge	Acknowledgement of at least one of the following: • Learning involves the integration of new ideas into a framework • Knowledge can be built by the student • Using your prior knowledge to understand new ideas • Always coded with student ideas, but the addition of constructing knowledge implies more action on the student’s part Use of the word constructivism	“Learning means to build upon knowledge that you already have a foundation.” (Spring 2014) “…integrate the information into their own mental models and work through the material themselves.” (Spring 2015) “In this process, you form connections between what you already know and new ideas.” (Fall 2015)
Formative assessment	Must include at least one of the following: • Use of the word “formative assessment” • Reference to building at least two parts of “the bridge” which consists of the following components: • Where the student starts (with respect to a given topic) • Where the student needs to go • How to help them get there Acknowledgement of the importance of finding out *why* your student does not understand	“Learning required small steps so that it can get us to where we ultimately want to go.” (Spring 2014) “It’s meeting them halfway across the ‘how we gonna get there’ bridge.” (Spring 2014) “Effective teachers engage in formative assessment through evaluating the level that their class currently is at (through examining test performances, clicker questions, etc.) and the level that the teacher wants them to be at, and coming up with methods of how to get their class there.” (Fall 2014)

Finally, we developed subcodes for our student ideas code, which we called *resource*, *obstacle*, and *vocabulary*. These codes largely emerged inductively from our data, as we tried to refine our understanding of the student ideas language present in LAs’ responses. However, we found that the subcodes we generated aligned with prior literature (e.g., Hammer 1996), and thus we clarified the definitions of these codes by building on prior studies.

We must acknowledge that the pedagogical principles were challenging to objectively code, and many iterations of coding were required to achieve definitional guidelines and clarity and reliability. We discuss some of our difficulties with particular codes below.

#### Student ideas

When coding for *student ideas*, we were looking for acknowledgement of at least one of the following statements: (a) students come in with ideas, (b) these ideas can impact the way the new information is taken up by students, (c) these ideas can be built upon or related to when teaching new information, or (d) the new information may cause some kind of rearrangement of a pre-existing framework. These four statements become progressively more complicated, for example, student idea code (a) simply requires some vocabulary use around student ideas, while (b), (c), and (d) require that LA acknowledged the *use* of student ideas in some way.

Certain words presented a clear picture (to us) of how the LA was thinking about student ideas. Such words or phrases included “prior, already know, mental model, pre-conceived notions, misconceptions, belief, old understanding, existing knowledge, previous knowledge, and student ideas.” We found that these words could be easily aligned with our code definitions and were thus ideal for standardizing our coding process. Examples of responses coded as *student ideas* can be seen in Table 2.

When coding for *student ideas*, it was often unclear whether LAs thought that students came into the classroom with ideas of their own or if the LAs thought that students’ ideas were simply regurgitations of ideas they were presented with in class. Also, if an LA assumed that whatever was taught in a previous or current class was taken up exactly as the instructor intended, statements about “prior knowledge” present a gray area. We resolved this issue by developing a coding rule that there must be a clear acknowledgement of ownership by the student of the ideas or there must be a clear acknowledgement that the ideas pre-dated the class. For example, the following responses were coded as *student ideas*:

“To help students understand the knowledge that they already have…” (LA reflection, Spring 2016)

“Learn what the student thinks and how they formulate ideas.” (LA reflection, Spring 2016)

In both of these responses, we see ownership and/or an indication that the student ideas were held prior to their interaction with the LA. By using the phrase “they already have,” the first LA indicates possession of the knowledge by the student and also that the knowledge was held by the student before the class. In the second example, although there is no obvious timeline, by saying “they formulate ideas,” the LA seems to be attributing the ideas to the student and not indicating that the student is just repeating the ideas they were given by someone else.

There were also some words used by LAs when referring to student ideas or their use that made coding difficult, such as “expanding, experience, grow(th), develop, opinions, information, thought process, real lives, level, shaping, or frame of mind.” These words were particularly difficult to code because it was not always clear what was meant by them, for example, whether the LA was crediting the student with authorship and control over their ideas or whether the LA was speaking in a more “acquisition-like” framework (Sfard 1998). For difficult-to-code responses such as these, we relied heavily on the context of the statements, making judgments that were later checked between the two coders.

##### Student ideas subcodes

We further divided the *student ideas* code (which was coded most often) into three subcodes: *resource*, *obstacle*, and *vocabulary* based on our own observations during the coding process. The purpose of these subcodes was to see how LAs were taking up the notion of students’ ideas with respect to their use in teaching and learning. We were interested in not only their recognition of student ideas but also in their ability to see them as a resource for learning.

We coded as *resource* when we saw evidence of LAs’ recognition of the benefit and usefulness of student ideas in LAs responses to the teach prompt or the learn prompt. An example of a resource-framework can be seen in the following definition of teaching:

“To help students understand the knowledge that they already have and how to *use* that information to understand new topics and concepts.” (LA reflection, Spring 2016, emphasis added)

Here, we noted that the LA was implying a clear use for a student’s prior knowledge in helping the student comprehend new ideas, and thus this response was coded *student ideas as resources*.

We coded *obstacle* as a recognition of student ideas that were viewed as something that needed to be overcome or changed to accomplish teaching and/or learning. For example, the following response demonstrates LAs’ views of *student ideas as obstacles*:

“I think that learning is the integration and application of knowledge within a pupil. The integration aspect depends on the teacher recognizing the learners current misconceptions and dispelling them with the new accurate knowledge.” (LA reflection, Spring 2014)

In the statement above, while the LA clearly acknowledges that a student has ideas when learning, the LA also implies that those ideas need to be moved out of the way to allow for new ideas to take their place. Thus, this response was coded *student ideas as obstacles*.

Finally, we coded *vocabulary* where we saw a recognition of student ideas, but there was no apparent value assigned to these ideas, and no explicit use of the ideas was recognized or mentioned. Most often, this coincided with the use of the term “mental models,” hence our choice of the “vocabulary” code descriptor. However, there were a few other instances where we found LAs acknowledging that students had ideas without explicitly identifying their functionality or usefulness and without using the mental models descriptor. We felt that such answers contained vocabulary consistent with language used in the course to discuss student ideas. Thus, we justified including these types of responses in the vocabulary category if they did not reflect a resources or obstacle perspective on student ideas. An example of this can be seen in the following excerpt:

“To teach is to bring students to a further point of knowledge than they began with. If a teacher can instill a sense of learning and interest in a subject, they know how to teach.” (LA reflection, Fall 2015)

While this response acknowledges that students begin with some knowledge, it assigns no particular value to this knowledge relative to its use in teaching or learning, and thus we conservatively coded this response *student ideas as vocabulary*.

We note that LA responses could occasionally be double-coded with these subcodes if one part of their response indicated that they were thinking about *student ideas as obstacles*, while in another section of their response, they demonstrated thinking about *student ideas as resources*. We argue that this is acceptable because any ideas that LAs have throughout the course are likely to be fusions of different concepts and language since they are still learning. An example of a double-coded response follows:

“Learning is listening to, actively-engaging with, and gaining new or different knowledge, integrating that knowledge into your current mental model, addressing any conflicts or misconceptions that arise and understanding the gained information.” (LA reflection, Spring 2014)

Here, we observe the LA viewing the integration of new knowledge with current knowledge as a critical resource for learning; however, they also use “misconceptions” terminology which implies more of an obstacle framing of student ideas. Thus, the above response was coded as *student ideas as resources* and *student ideas as obstacles*.

#### Constructing knowledge

Our second code of interest for this study was *constructing knowledge*. When coding for *constructing knowledge*, we were looking for acknowledgement of at least one of the following ideas: (a) learning consists of the integration of new ideas into a framework, (b) knowledge can be built by the student, (c) students use prior knowledge to understand new ideas, or (d) use of the word constructivism. We note that responses of type (c) were always double-coded with *student ideas*.

For category (a), we struggled with whether or not the “framework” that new ideas could be integrated into necessitated double-coding with *student ideas*. For the most part, we found that the *student ideas* code was implied by these responses, but occasionally the framework mentioned by LAs was too vague to definitively code *student ideas* as well. For example,

“To learn is to construct a world view in which one intakes and accept knowledge that ‘works’ and rejects knowledge that doesn’t work.” (LA Reflection, Spring 2016)

In this response, a framework was implied as a way to accept or reject knowledge, but we did not see explicit mention of student ideas. Thus, this response was solely coded as *constructing knowledge*.

Similar to our coding of *student ideas*, we uncovered some problem words when coding for *constructing knowledge*, namely, “expanding, experience, grow(th), develop, exploration, thought process, shaping, learning through discovery, applying, connecting, and information.” We found that these words could be interpreted in two different ways, with one empowering the student to build their own knowledge (as per constructivism) or with a student being led to information (more of an acquisition lens). Our coding of these terms became very context dependent. We did find that there were some consistently clear words that indicated *constructing knowledge* such as “building, integration, creating meaning, and construct.” Whenever these terms appeared in responses, we found clear evidence of a constructivist framework.

#### Formative assessment

When coding for *formative assessment*, we were looking for responses that included at least one of the following: (a) use of the phrase “formative assessment”; (b) demonstration of building the bridge (as described in the pedagogy class); (c) acknowledgement of where the student starts, where they need to go, and how to help them get there with respect to content; or (d) mention of finding out *why* a particular student does not understand a concept. For the building of the bridge in part (b), we noticed that several students specifically referred to the bridge picture that we used to introduce formative assessment in the pedagogy class. For responses including the bridge to be coded, we required that the language used be student-focused (i.e., not about the teacher improving their practice) and that LAs included at least two components of the bridge in their response (e.g., where the student starts relative to where they need to be or where the student starts and how they could be guided down a path in the right direction).

We found *formative assessment* particularly challenging to code given the number of components necessary to create the full formative assessment picture. It was difficult to decide at what point an LA was beginning to use formative assessment language even if they had not developed the full concept yet. In particular, we found that students began using some goal-setting language, which mimicked the language in the formative assessment article we distributed (Moss and Brookhart 2010), but there was no acknowledgment of student ideas. For example,

“You set a goal of where you want your students to be by the end of the course, and the way in which you teach them will help them get to the goal or not.” (LA Reflection, Fall 2014)

To resolve this, we decided that any response that incorporated two components of the bridge, even if it did not acknowledge where students were starting from, was indicative of early stages of an LA expressing the concept of formative assessment and thus goal setting language was coded as a formative assessment if the response indicated a goal and a path to achieve the said goal. Additionally, some LAs did not describe a complete bridge, even though they acknowledged the student’s starting place and the desired ending place.

“As a teacher it is important to understand where your students are coming from and where they need to go in terms of their understanding.” (LA Reflection, Spring 2015)

Again, we saw two components of the bridge here and thus decided that this response and similar responses were indicative of the early stages of expressing formative assessment.

We also discovered that finding *formative assessment* in responses to the “What does it mean to learn?” question was rare. Instead, LAs seemed to rely more heavily on metacognition as a way to check in on learning progress with respect to this question. For example,

“This [learning] includes: having an open mindset, assessing your current knowledge and knowledge gained periodically through the learning process, and reflecting on your own studying and learning techniques to determine what strategies are the most effective for you.” (LA Reflection, Spring 2016)

Though we were not specifically coding for metacognition in the study reported here, based on the patterns seen in LA reflections as well as our informal observations of LAs during class discussions, it seemed that the language associated with this concept (which we also discuss in the pedagogy course) was being taken up as the learning analog of formative assessment in teaching. However, metacognition was not one of the principles we focused on in this study, and thus we excluded such responses from our *formative assessment* coding.

#### Reliability

To ensure the reliability of this study, all responses were coded independently by two different researchers, one semester at a time. After analyzing a given semester, the researchers came together to resolve any conflicting codes. We found that the resulting number of code changes was minimal, only 1.5% for student ideas, 1.2% for constructing knowledge, and 0.4% for formative assessment. After resolving any conflicts, we developed formal definitions for each of the codes and then analyzed all responses again. We found that this also resulted in a small number of changes to the code counts. Although the coders were instructors for sections of the pedagogy course for two of the semesters included in this study, section numbers and names were blinded during coding to minimize the potential for researcher bias.

#### Limitations

We acknowledge that this study is restricted in scope and thus has several limitations. First, this research is meant to serve as a beginning assessment of what pedagogical content students (LAs) learned while enrolled in a course about pedagogy. It is intended to mirror studies that seek to evaluate learning of any content (e.g., physics) by comparing pre- and post-measures of students’ understanding. This study is not intended to determine the extent to which LAs used these ideas in practice, although such a study could be warranted, and in fact, is taking place.

We also acknowledge that a validity argument could have included member checking with previous LAs; however, this would not have been practical given the span of the data and the numbers of subjects. We took great care to operationalize our codes and coding process to make conservative estimates of the meaning being made by participants in the study.

Additionally, our initial review of the data revealed that LAs’ reflections included large numbers of references to student learning as acquisition (Sfard 1998) rather than construction. An inductive coding scheme could have provided more nuanced insights into what LAs take away from the course and the LA experience. However, the purpose of this study is to investigate the extent to which LAs took up the pedagogical concepts addressed in the pedagogy course as discussed previously.

Finally, in attempts to find an efficient measure for assessing LAs’ understanding of the pedagogical principles that were taught in class, we asked LAs general questions, “what is teaching and what is learning.” Our inductive coding allowed us to establish coding rules that accurately reflected LAs’ ways of applying each pedagogical principle to these general questions. The coding rules provide insight into the ways LAs were thinking as well as the extent to which their thinking was different pre- and post-instruction. The resulting graphs shown in the following section provide visualizations of the distributions of these responses, allowing for inferences about LAs understanding of pedagogical principles. While our ultimate goal is to understand how much LAs learn throughout their LA experience, this work is purely descriptive and we are not trying to make causal claims about the growth of LAs as a result of the pedagogy course. The coding percentages presented in the figures and findings are intended to lend insight into trends in LAs’ responses, which are exemplified in the methodology section. Our analysis is largely qualitative, illustrating trends in LAs’ responses to a very difficult, abstract question, intended to assess deep understanding of a concept.

### Findings

**Fig. 3:**
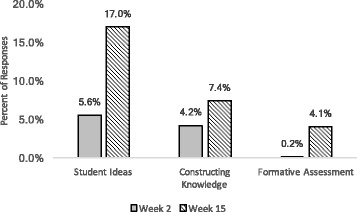
Averaged overall major codes. The percentage of responses that were coded as *student ideas*, *constructing knowledge*, and *formative assessment* and averaged across five semesters. Week 2 responses are shown in solid gray, while week 15 responses are indicated by the striped bars

To address our primary research question, (to what extent did LAs refer to essential pedagogical principles in written responses to questions about teaching and learning?), we calculated the percentage of responses to both the teach prompt and the learn prompt that were coded *student ideas*, *constructing knowledge*, or *formative assessment* each semester and then averaged them across the five semesters in our study (Fig. 3). In almost all cases, LAs answered both the teaching and the learning questions, so there were approximately two responses per LA for week 2 and two responses per LA for week 15. Since there were five LAs that discussed their views of teaching but did not respond with their definition of learning in Spring 2016, the results are reported in terms of percentage of responses instead of a percentage of students.

In week 2, a few LAs came into the pedagogy class with some constructivist or student ideas language already; about 4% of LA responses demonstrated some ideas about constructing knowledge, while 6% of LA responses indicated that students have pre-existing ideas. We did not see any indications of LAs using or understanding formative assessment in week 2 responses. For all three concepts, we saw positive growth on average throughout the semester. Our data indicates that the LAs showed the most growth around student ideas, with an 11% increase in the number of responses coded as *student ideas* between weeks 2 and 15. Growth was seen in *constructing knowledge* and *formative assessment* as well, with 3 and 4% increases in responses coded as these concepts, respectively.

### How the language associated with the concept of student ideas is taken up by LAs

**Fig. 4:**
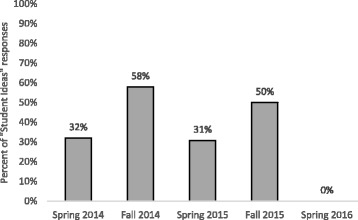
Mental models term usage. Percentage of responses to the teach and learn prompts that were coded as *student ideas* that also contained the term “mental models”

Since LAs demonstrated the most growth with respect to the concept of student ideas, we analyzed the responses coded as *student ideas* in more detail. In many responses, we noticed that LAs were specifically using the term “mental models” which was a key word in the Redish (1994) reading handed out in four of the five semesters (the article was not assigned in Spring 2016). In fact, in the four semesters that the Redish article was distributed, between 30 and 60% of the responses that were coded as *student ideas* in week 15 explicitly contained the term “mental models” (Fig. 4).

While these numbers vary among the four semesters, it is clear that at least 30% of LAs who indicated the importance of student ideas in their responses also took up the term mental models in their thinking about the concept each semester. Note that there are no mentions of mental models in Spring 2016, the semester that we did not assign the Redish article. This indicates that the mental models language was likely coming from the article distributed in the pedagogy course and not from some external source. Figure 4 also shows that the mental models language seems to be greater in the Fall than in the Spring semesters.

**Fig. 5:**
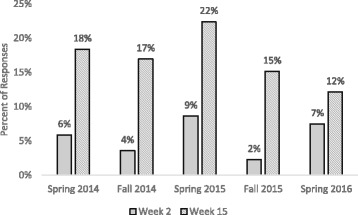
Student ideas codes by semester. Percentage of responses coded as *student ideas* in week 2 (solid gray bars) versus week 15 (patterned bars) from Spring 2014 through Spring 2016. These responses include both LAs’ definitions of teaching and learning

By investigating the breakdown of *student ideas* codes by semester, we determined how the growth in student ideas aligned with LA use of the mental models language. For each of the five semesters, we looked at the percentage of teaching and learning responses coded as *student ideas* in week 2 relative to the percentage of responses coded as the concept in week 15 (Fig. 5).

Though the number of LAs coming in with student ideas language varies quite significantly across the semesters, the growth from week 2 to 15 is around 12–13% of responses each semester except for Spring 2016 where we only see a 5% growth. This seems to be correlated with the absence of a mental models article in that semester. Note that the growth in student ideas codes does not necessarily follow the same pattern as mental model usage across Fall versus Spring semesters. While we saw that mental model language was consistently used in responses coded as *student ideas* from Spring 2014 through Fall 2015, the take-up of the term was particularly high in Fall 2014 and Fall 2015 (58 and 50%, respectively). However, the growth in the number of responses coded as *student ideas* is nearly invariant (around 12 or 13%) for all four of the semesters in which the Redish article was used, that is, we do not see substantially higher growth during the Fall semesters. Thus, it seems that an increased use of mental models terminology in a given semester does not correlate directly to more growth in student ideas codes during that semester. Only when there is a complete absence of mental models language do we see a change in student ideas growth; the number of student ideas codes decreases dramatically in Spring 2016 when the term mental models were not introduced in the pedagogy course. However, even though LAs were not using the term mental models explicitly as frequently in Spring 2014 and Spring 2015 as in the Fall semesters, the ideas and language in the article may have still facilitated discussion around student ideas, leading to the same amount of growth as in other semesters.

### How deeply the language associated with the concept of student ideas is taken up

Our data indicated that LAs were beginning to use discourse around student ideas, but our initial coding did not indicate how deeply that learning went. We wanted to see if LAs actually found student ideas useful in teaching and learning. In order to investigate this, we took two analytical approaches to measuring the sophistication of LAs’ responses: first using the resource framework described by Hammer (1996) and then analyzing in terms of the formative assessment scale introduced by Gray (2013).

#### Is the language related to student ideas taken up in a resource framework?

**Fig. 6:**
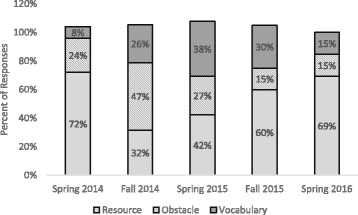
Breakdown of student ideas codes. Percentage of week 15 teaching and learning responses coded as *student ideas* that talked about *student ideas as a resource* (light gray), *obstacle* (patterned), or simply used *vocabulary* (dark gray)

We looked at the distribution of responses coded as *student ideas* that used resource or obstacle language surrounding student ideas as well as responses that were simply using the vocabulary without putting any value on the student ideas (as described previously in our definition of the *student ideas as vocabulary* subcode). This distribution is shown for each semester from Spring 2014 to Spring 2016 (Fig. 6).

Note that the columns do not sum to 100% for every semester because some LA responses were double-coded as *resource* and *obstacle* as explained in the “Methodology” section. The data indicates great variation in the ways that LAs talked about student ideas across the semesters. For Spring 2014, Fall 2015, and Spring 2016, over half of the student ideas were talked about using resource language. In fact, on average across all semesters, 55% of *student ideas* responses used resource language. Note that a 55% average of *resources* coded within responses already coded as *student ideas* actually amounts to only 9% of total LA responses. In Fall 2014, however, obstacle language was more dominant, and in Spring 2015, there was a fairly even distribution between all three subcodes. If we define “deep” learning to mean obstacle or resource language (i.e., referring to student ideas in a more than superficial way), we see that over 60% of LA responses that discussed student ideas demonstrated deep learning.

#### Comparing LAs’ learning of student ideas

**Fig. 7:**
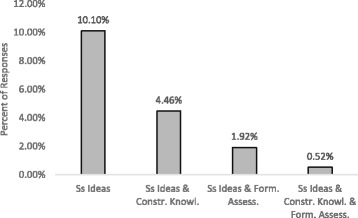
Averaged week 15 coding percentages. Percentage of week 15 student responses coded as *student ideas* in conjunction with *constructing knowledge* and *formative assessment*. Note that the columns are mutually exclusive

Another way that we assessed the depth of LAs’ learning regarding student ideas was by looking at *student ideas* codes in conjunction with *formative assessment* and *constructing knowledge*. We argue that if the value of student ideas is truly learned, this will lead LAs to a better understanding of the latter concepts because they represent the positive utilization of these student ideas in the teaching and learning processes. To that end, we looked at the percent of week 15 responses that were coded with only *student ideas*, double-coded with only *student ideas* and *constructing knowledge*, double-coded with only *student ideas* and *formative assessment*, or triple-coded with all three. These percentages were then averaged across all semesters (Fig. 7).

From this graph, we see that approximately 10% of responses were only coded as *student ideas*, and the percent of responses that were coded as *student ideas* in conjunction with either of the other concepts was 5% or less. This seems to indicate that even though LAs begin to take up the language of student ideas throughout a semester in the pedagogy course, they do not necessarily extend that concept to constructing knowledge or formative assessment.

## Discussion

This study sought to create an efficient measure of the learning of selected pedagogy course content through a written pre/post-assessment. Our data clearly show that out of our three essential pedagogical principles, the language related to student ideas is most likely to be taken up by LAs over the course of a single semester. Why are LAs more likely to develop the language of student ideas than that of constructing knowledge or formative assessment? Arguably, “student ideas” is a topic that is intentionally woven through many weeks of the pedagogy course, which means that LAs are likely to get more discussion time and more reinforcement of the importance of student ideas than some of the other concepts. However, we also hypothesize that student ideas is a more relatable and tangible concept for LAs to grasp. Words like constructivism and formative assessment can seem strange and unfamiliar to LAs, and they may have few prior ideas to relate to this terminology. Student ideas, on the other hand, does not contain any jargon that may create a barrier for LAs connecting to the terms. Additionally, LAs can connect student ideas to their own experiences as students. Also, the concepts of formative assessment and constructing knowledge require an understanding of student ideas, thus student ideas may be more fundamental and easier to learn.

We also hypothesize that the term “mental models,” through which the notion of student ideas is introduced, seems to resonate with LAs. This language may be relatable to our math and science LAs who are often familiar with the idea of models from their own math and science disciplines. For example, chemistry students are often introduced to different models of the atom which become progressively more refined as new ideas are introduced that contradict prior assumptions or reveal new data. This may give LAs a way to think about student ideas being refined with new evidence and integrated into prior frameworks. The language of mental models and misconceptions have been gradually avoided by the education community due to the connotation of students being “stuck” in a particular way of thinking, the negative framing of students being wrong, or the connotation of a mental model being long-lasting and invariant. In science and math, however, models are often seen as valuable temporary approximations that continue to improve with further evidence. Further, the mental models framework provides LAs with a familiar mechanistic approach to learning that aligns with how LAs may think about growth and change. Thus, LAs may be drawing on their own prior knowledge of models in order to build an understanding of the role of pre-existing ideas in learning.

Our data suggest that the take-up of mental models varied greatly in the semesters in which the phrase was introduced. The mental models language was used in approximately 30% of student ideas responses in Spring semesters (2014 and 2015), while it was used in 50% or more student ideas responses in Fall semesters (2014 and 2015) as shown in Fig. 4. The reason for the variation between Spring and Fall semesters is unknown, but we hypothesize that this has to do with having more second-semester freshmen in the Spring semesters and fewer freshmen and more upper-class LAs in the Fall. Thus, there may be less “mental models” language development because freshman LAs may not have strong model-based frameworks from science courses on which to build. We also have different courses across the departments that use LAs in Fall and Spring, though it is not clear how this might impact the development of language in the pedagogy course. Even though the take-up of mental models terminology is varied, we see consistent growth in student ideas. Again, this may be due to LAs in the Spring semester having just completed the course they are LAing for themselves, with only a short winter break, thus having their own ideas as students fresh in their minds. In addition, they may see themselves more similar to the peers they are assisting since they are more likely to be in a comparable place in their education. They may, therefore, more easily recognize the value of students’ ideas, leading to the same conceptual growth.

LAs’ learning of the student ideas concept went beyond vocabulary. As indicated by the data, LAs discuss student ideas as resources or obstacles as well. These three ways of talking about student ideas are consistent with Hammer’s (1996) description of the three frameworks with which pre-service teachers in his study view student ideas. We found that on average, half of LAs’ responses that discuss student ideas are framing them as resources for teaching and learning, which indicates learning that is deeper than just vocabulary usage and in fact, even deeper than an obstacle or misconceptions view. Despite the fact that we only see 12–13% growth in responses that discuss student ideas, most of those responses view student ideas as critical for the learning process (whether as resources or obstacles), which we feel is a significant development in LA language, for only one semester of instruction in a formal pedagogy course.

Although LAs’ language indicated that they began taking up student ideas as resources, we also found that their learning did not extend to taking up the concepts of constructing knowledge and formative assessment. It makes sense that an understanding of the value of student ideas would come before an understanding of constructing knowledge and formative assessment since student ideas are foundational for both of these concepts. Furthermore, we hypothesize that unlike student ideas and mental models, the terms “constructivism” and “formative assessment” may not resonate as much with this population (though the associated language may be more readily taken up in other contexts, such as teacher education programs). Although the concept of constructing knowledge has always been a part of the pedagogy course, the term was only formally introduced with a reading on “constructivism” during Spring 2016. The term may not have been relatable to LAs during their LA experience, yet without a term, the concept may be difficult to grasp. Similarly, LAs struggled to connect with the term “formative assessment.” Assessment likely cues images of tests for LAs, making it difficult for them to distinguish between formative and summative assessment. Formative is also a problematic word, since LAs often have little prior exposure to this word. In Fall 2016, we began using the word “bridging” in place of formative assessment in hopes of relating the concept to the three-component bridge that we use to introduce the topic in the pedagogy course, as well as relating to a practical object that LAs likely have prior exposure to. We suggest that the use of this new terminology will facilitate better uptake of the concept of formative assessment, and our preliminary observations (though not quantified) seem to support this hypothesis. We are regularly revising course components in response to research findings and the changing student population. However, as we make changes, it is critical that we hone in on a systematic method for evaluating the learning of pedagogy course content, as described in this paper, in order to measure the impact of our modifications. Additionally, it is critical to understand which outcomes are of value, to whom, and under what circumstances.

The reader may be surprised that the actual differences between pre- and post-use of the essential pedagogical principles were relatively low on average. Few studies actually measure the uptake of pedagogical principles; therefore, we have little means for comparison (e.g., Rosebery 2015). There are several possible reasons that learning gains were low as well as important implications. One possible explanation for the minimal gains may be that pre/post-semester measures assume that learning takes place during this relatively short period of time. It may be that learning pedagogical principles take much more time and practice. Smagorinsky et al. (2003) discussed this phenomenon with respect to teacher learning; they assert that prolonged engagement with pedagogical principles through practice is necessary for deep learning. Furthermore, LAs often participate in multiple learning environments, which may offer conflicting models of teaching and learning. These models, which are often in opposition to the concepts introduced in the pedagogy course, may also inhibit LAs’ development of the pedagogical principles language.

Another explanation for the small gains observed may be that pedagogical language development is simply difficult to measure through written forms of assessments. The data and results presented here certainly do not reflect the level of pedagogical sophistication that we have observed when working with LAs. It may be that first-semester LAs are in the more nascent stages of pedagogical development, which may limit our ability to accurately assess concepts such as formative assessment by analyzing their response to a general prompt on definitions of teaching and learning. Developing operational, abstract definitions of principles is often a different task than developing practical, contextually bound implementation of the concepts (Vygotsky 1986). In other words, our current methods and the data analyzed here may not have been optimized to detect the current sophistication of first-time LAs’ pedagogical knowledge.

## Conclusions and Future work

Future work will attempt to optimize the detection of LAs’ current understandings, and we hypothesize that a revised approach will reveal much greater LA development than was uncovered in our current analysis. Indeed, we have found in our own preliminary work that LAs’ written responses to contextualized prompts were more substantive than for the prompts analyzed here, which were more abstract and required the formulation and application of a general principle, such as formative assessment. In fact, LAs’ responses to a prompt (not included in the pre/post-prompts that were the subject of the current study) indicated that LAs use language associated with formative assessment when asked directly. For example, one of the reflection prompts specifically asked LAs what they would say if they discovered that one of their students had an idea that was not fully correct. This led to answers such as those shown below.

Example 1: “I would try to backtrack to the point where the student does have the correct ideas about the topic, even if I had to go back to the very fundamentals of the topic. And then once that baseline was established, I would try to build on that knowledge using constructivism and bridging to work back up to where the concept was discussed in class the previous day.”

Example 2: “I would ask them to explain to me what their understanding of the topic is, and ask leading questions as they do so to help them (and me) understand where their ideas might not be totally correct. From there I would build on the greatest common denominator, encouraging them to use any correct information base in the topic they might have as a foundation, and build on that or bridge from that to where they need to be.”

In each of these examples, the LAs seem to demonstrate an understanding of formative assessment that goes beyond language use. Both LAs refer to “bridging” explicitly (the language that was used to introduce formative assessment in this particular semester) but beyond using the vocabulary; they describe pedagogical principles such as eliciting student ideas and helping students construct their own knowledge using strategies like questioning in order to reach a goal. These components are illustrated in the LAs’ responses to a situational prompt indicates that LAs do in fact have a much better grasp of the concept of formative assessment than indicated in their more abstract definitions of teaching and learning.

In preliminary studies of LAs in action, members of our research team found the four main LA actions observed (over four different LA settings in four different departments) were teaching with formative assessment, direct instruction, establishing comfort, and sharing study strategies and resources. When LAs were observed teaching with formative assessment, they generally checked for comprehension, elicited student thinking, or asked guiding questions or provided advice based on students’ thinking.

It appears that being able to operationalize the concept that students have prior knowledge (students ideas code) may be an important threshold that allows LAs (or early-career teachers, for that matter) to interact effectively with students in the classroom, in ways that look very much like formative assessment. Certainly, more work is necessary for revealing the actual role that LAs are playing in impacting student success as well as in figuring out robust and efficient ways of measuring the learning of pedagogical principles.
